# The Interplay Between Circadian Clocks and the Tumour Microenvironment in Breast Cancer

**DOI:** 10.3390/cancers18060925

**Published:** 2026-03-12

**Authors:** Anna-Marie Finger, Carolin Ector, Valerie M. Weaver

**Affiliations:** 1 Obesity Discovery, Global Research, Research & Development, Novo Nordisk A/S, 2880 Ballerup, Denmark; aqnf@novonordisk.com; 2 The Francis Crick Institute, 1 Midland Rd., London NW1 1AT, UK; carolin.ector@crick.ac.uk; 3 Center for Bioengineering and Tissue Regeneration, Department of Surgery, University of California, San Francisco, 513 Parnassus Avenue, HSE 565, San Francisco, CA 94143, USA; 4 Department of Radiation Oncology, Department of Bioengineering and Therapeutic Sciences, Eli and Edythe Broad Center of Regeneration Medicine and Stem Cell Research, Helen Diller Family Comprehensive Cancer Center, University of California San Francisco, San Francisco, San Francisco, CA 94143, USA

**Keywords:** circadian clocks, circadian rhythm, cancer, breast cancer, extracellular matrix, stiffness, mechanotransduction, metastasis, mechanobiology

## Abstract

Cancer is a heterogeneous systemic disease that is strongly influenced by dynamic interactions with the tumour microenvironment (TME). Despite major advances in understanding spatial and molecular tumour heterogeneity, the temporal dynamics of tumours have received far less attention. Growing evidence has linked circadian clocks to cancer risk, progression, and treatment response, including in breast cancer. However, temporal regulation has yet to be recognized as a cancer hallmark, and its interaction with the TME remains poorly understood. This review examines how circadian rhythms organize breast cancer biology through bidirectional interactions with the TME. Circadian clocks coordinate proliferation, DNA damage responses, metabolism, and immune surveillance. Ageing, chronic stress, and obesity, all of which are established breast cancer risk modifiers, disrupt these rhythms and are reciprocally exacerbated by circadian dysfunction, establishing feed-forward loops that accelerate disease. Within the TME, the extracellular matrix (ECM) plays a central role in mediating this bidirectional control. Stiffened fibrotic stroma dampens epithelial clock amplitude, while circadian rhythms in turn shape collagen turnover and ECM remodelling. These dynamics can foster inflammation, stem cell expansion, and metastatic dissemination, including time-of-day-dependent release of circulating breast tumour cells. Systemically, circadian clocks gate immune cell trafficking, creating predictable windows of immunosurveillance and therapeutic vulnerability. By integrating insights from mechanobiology, metabolism, immune regulation, and ageing, we position circadian timing as a unifying layer that connects cell-intrinsic programmes with the evolving breast TME. Understanding these connections opens new opportunities for chronotherapeutic strategies in which treatment timing is aligned with circadian rhythms to improve outcomes.

## Simple Summary

This review explores how the body’s internal 24 h rhythms, driven by circadian clocks, interact with the microenvironment surrounding a tumour (called the tumour microenvironment (TME)), specifically in breast cancer. While the physical and molecular aspects of cancer are well understood, its temporal dynamics have received less attention. The review outlines how circadian rhythms coordinate crucial cellular biological processes like proliferation, DNA repair, metabolism, and immune surveillance, and how factors such as ageing, chronic stress, and obesity can disrupt these rhythms, contributing to cancer progression. The review extends to a discussion of how the TME, and specifically the biochemical and biophysical properties of the extracellular matrix (ECM), act as a central component mediating this bidirectional control between cell-autonomous rhythms and pro-tumorigenic changes. By understanding these complex temporal connections, the research community can develop new “chronotherapeutic” strategies to reduce the risk of malignancy and time treatments to align with circadian rhythms to improve patient outcomes.

## 1. Breast Cancer in Context

Cancer is a complex and heterogeneous disease that arises in and can disseminate to multiple tissues [1]. Breast cancer in particular is a solid tumour that ranks second in global incidence, with 2.3 million new cases and 670,000 deaths reported in 2022, and with its incidence projected to nearly double by 2070 [2,3]. Tumour development involves the gradual accumulation of genetic and epigenetic alterations that disrupt normal cellular function [1]. In 2000, six fundamental “Hallmarks of Cancer” were proposed, including autonomous growth signalling, resistance to growth suppression, evasion of apoptosis, limitless replication, sustained angiogenesis, and the ability to invade and metastasize [4]. Many of these tumour cell intrinsic hallmarks are variably expressed in the malignant cells, including in the different breast cancer subtypes [5].

Over the past few decades, there has been a growing appreciation for the importance of the cellular and non-cellular tumour microenvironment (TME) in cancer progression [6,7]. The TME consists of a cellular fraction that includes fibroblasts, smooth muscle cells, endothelial cells, and pericytes that comprise the stroma and vasculature, as well as adipocytes, nerves (sympathetic, parasympathetic), and a highly heterogeneous and evolving immune component [8,9]. The cellular TME is highly heterogeneous and has been shown to play a critical role in malignant transformation and metastasis of the breast, as well as in its treatment [6,10]. Recognition of the importance of the cellular TME led to the expansion of the hallmarks by two TME traits, namely evasion of immune destruction and tumour-promoting inflammation. Furthermore, two tumour cell-specific traits were incorporated into the updated framework: reprogramming of energy metabolism, as well as genome instability and mutational burden [11]. Importantly, these new cancer hallmarks are heterogeneously expressed in many cancers, including breast cancer [12].

A less appreciated component of the TME, yet a critical one for cancer pathogenesis and treatment response, is the non-cellular TME component comprising soluble factors—such as growth factors, morphogens, chemokines, and cytokines—exosomes, and the extracellular matrix (ECM). Of these non-cellular TME components, the ECM is now recognized as an important modifier of malignant transformation, tumour progression, and treatment response [6,7]. Emerging data have revealed how the tumour ECM influences multiple breast tumour cell phenotypes, including growth, survival, migration, and invasion [13,14,15,16,17,18,19]. The ECM can also modify the function of the cellular constituents of breast tissue to drive malignant transformation and metastatic dissemination, as well as to modify therapeutic response [18,20,21,22]. Recognition of the key role of the ECM in cancer led to the further expansion of the tumour hallmarks to include the biochemical, biophysical, and organizational features of the ECM [23,24,25,26]. Critically, the tumour ECM itself is highly heterogeneous both with respect to biochemical properties as well as biomechanical features, implicating this specific noncellular tissue component in tumour development and evolution [25,27,28,29,30]. Indeed, emerging data underscore the importance of not only ECM composition but also its organization and mechanics, especially with regard to breast tumour evolution and aggression [25].

Cancer is both a tissue and systemic disease, characterized by spatiotemporal dynamics that drive cancer initiation, progression, and dissemination [31]. One temporally regulated entity known as the circadian clock has been implicated in tumour progression and associated with breast cancer risk, development, and treatment response [32,33,34,35]. The circadian clock is an autonomous, internal timekeeping system that influences a range of systemic responses as well as multiple physiological behaviours at the tissue level by altering cellular responses, including signalling and gene expression [36,37]. Recent studies have expanded these effects to reveal strong correlations between circadian clock regulation, anti-tumour immunity, and tissue mechanics, suggesting dysregulation of these parameters collaborates to compromise tissue homeostasis and promote cancer [38,39,40]. Ageing and chronic stress, contributing to circadian disruption, repress the immune response to increase breast cancer risk, promote tumour progression, and compromise treatment efficacy [32,41,42]. Nevertheless, despite compelling evidence supporting the importance of circadian clocks in modulating cancer risk, progression, and treatment, this parameter has yet to be incorporated into the cancer hallmarks classifications, and its interplay with the TME is habitually ignored. In this review, we discuss the hallmarks of cancer with a specific focus on breast cancer. We expand the hallmarks concept to incorporate the role of the circadian clock as it pertains to tissue homeostasis and its interactions with the epithelium and the TME (Table 1).

### Factors Impacting Breast Cancer Risk and Progression

Breast cancer risk is shaped by a combination of genetic, lifestyle, and hormonal factors. Approximately 15% of breast cancer cases are hereditary, often driven by mutations in the *BRCA1* and *BRCA2* genes, which are crucial for DNA repair [55,56,57]. Additional genetic variants in tumour suppressor genes, e.g., *TP53*, *PTEN*, *CDH1*, and *STK11*, contribute to hereditary breast cancer susceptibility, though with a lower incidence [58,59,60,61,62].

Molecularly, breast tumour classification relies on three standardized biomarkers, estrogen receptor (ER), progesterone receptor (PR), and amplified human epidermal growth factor receptor 2 (HER2) [63]: (1) Luminal A (Lum A) tumours (ER^+^, PR^+^, HER2^−^) are associated with a better prognosis, (2) Luminal B (Lum B) tumours display a less distinct profile (ER^+^, PR^+/−^, HER2^+/−^), typically resulting in a higher histologic grade than Lum A, (3) HER2-enriched tumours (ER^−^, PR^−^, HER2^+^) display a more aggressive course, (4) triple negative breast cancer (TNBC, ER^−^, PR^−^, HER2^−^), the basal-like subtype of breast cancer, accounts for approximately 15% of all diagnosed tumours and is usually of high invasiveness accompanied with a poor prognosis in regard to survival, recurrence, and distant metastasis [64,65,66]. Interestingly, high mammographic density with high percent stromal imaging opacity is characterized by an altered TME, including a stiffer stromal ECM, similar to that which develops in breast cancers, and appears to be a heritable factor defining up to 10% of the female population and increasing lifetime risk to developing breast cancer by up to fourfold [30,67,68].

Nongenetic risk factors exert pleiotropic effects on the TME. In fact, each of the breast cancer subtypes develops unique stromal phenotypes. While all breast cancers develop fibrosis and show some degree of immune infiltration, the higher grade HER2^+^ and TNBCs exhibit the most profound fibrotic response, and are characterized by stromal fibroblast proliferation and myofibroblast differentiation that promote a high level of collagen crosslinking, linearization, and stromal stiffening [27,69]. Breast tumour cells interacting with a stiffer stroma have elevated phosphoSTAT3 activity and express high levels of proinflammatory cytokines [70]. Consequently, these subtypes also have the highest levels of infiltrating immune cells, including CD8 T cells. Not surprisingly, TNBC, despite being the most aggressive subtype with extensive fibrosis and stromal stiffening, demonstrates the best initial responsiveness to immune checkpoint inhibitors [8]. At the systems level, ageing is also frequently accompanied by immune repression. The postmenopausal, aged breast has increased adiposity with concomitant inflammation and an altered ECM stroma that contains abundant senescent fibroblasts [71]. Moreover, luminal breast cancers are the dominant subtype diagnosed in postmenopausal women, whereas younger women typically develop TNBC or HER2^+^ breast cancers [72].

Lifestyle factors, such as chronic stress and obesity, shape breast cancer risk, subtypes, and disease progression [73,74]. For example, obesity is accompanied by fibroblast-myofibroblast activation and increased ECM deposition, turnover, and crosslinking that progressively stiffen the stroma and induce cytokine levels to increase local inflammation [75,76]. Additionally, obesity increases estrogen production via adipose aromatization and elevates ER^+^ breast cancer risk in post-menopausal women [77], while in pre-menopausal women, it is more strongly associated with the TNBC subtype through non-hormonal mechanisms, including immune dysregulation and metabolic alterations [74,78]. Chronic stress can increase breast cancer incidence through stress hormone-induced repression of anti-tumour immunity, modifications to the tissue stroma, and inflammation [79]. Under acute stress, glucocorticoids, such as cortisol, resolve inflammation by activating the glucocorticoid receptor (GR) [80]. Conversely, chronic stress induces GR resistance, resulting in elevated cortisol levels that fail to suppress inflammation effectively [81].

Intriguingly, chronic stress and obesity may also amplify breast cancer risk by disrupting circadian rhythms, although the precise contribution of circadian misalignment relative to other oncogenic mechanisms remains to be fully established. Prolonged glucocorticoid exposure by chronic stress alters the phase and amplitude of peripheral clocks [82,83,84], while obesity dampens hormonal rhythms and sustains inflammatory signalling that weakens circadian organization [85,86]. These processes reinforce one another: circadian disruption worsens metabolic and stress responses, which in turn further destabilize temporal coordination and create conditions that favour tumour development [87,88]. Shift work provides a real-world model of this interaction through repeated misalignment of behavioural and endogenous cycles, which promotes circadian disruption, stress, and metabolic dysfunction [89]. Consistent with this framework, shift work is associated with increased breast cancer risk [90], and tumours show altered clock gene expression compared with adjacent normal tissue [34,91,92,93,94,95]. Thus, chronic stress and obesity may shape breast cancer biology not merely through parallel mechanisms but through their convergent disruption of circadian coordination.

## 2. Circadian Clocks

Life on Earth has evolved in alignment with the planet’s 24 h rotation, leading to the development of internal timekeeping mechanisms [96,97]. The field of chronobiology traces its origins to the 18th century, when Jean-Jacques d’Ortous de Mairan and Carl Linnaeus demonstrated that plants maintain intrinsic rhythms independent of external light cues [98,99]. The molecular foundations of these observations were discovered in the late 20th century, where the key genes and proteins driving circadian rhythmicity in fruit flies were described and acknowledged with the Nobel Prize in Physiology or Medicine in 2017 [100,101,102].

**Figure 1:**
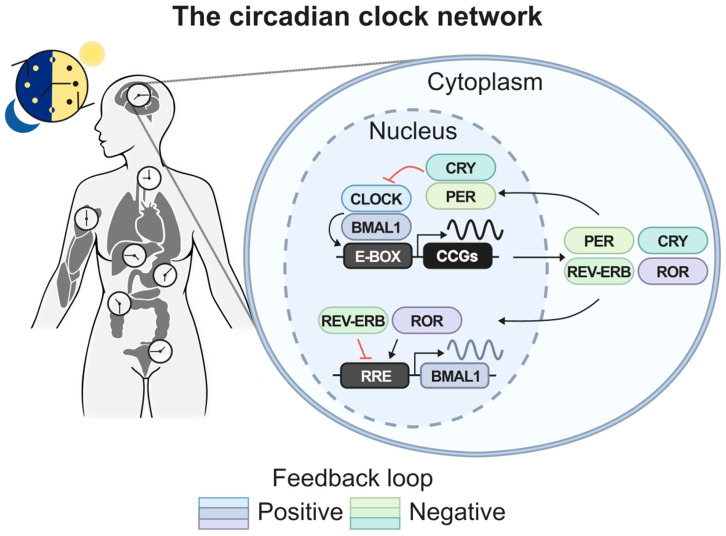
Organization of the mammalian circadian clock network. Circadian rhythms are coordinated across tissues and generated at the cellular level by interlocked transcriptional and translational feedback loops. In the nucleus, CLOCK and BMAL1 bind enhancer box (E-box) elements to drive rhythmic expression of *PER*, *CRY*, and other clock-controlled genes. PER and CRY proteins accumulate in the cytoplasm and translocate to the nucleus, where they inhibit CLOCK-BMAL1 activity, thereby forming a negative feedback loop. A secondary loop involves REV-ERB and ROR, which repress or activate BMAL1 transcription via RRE elements. Together, these positive and negative feedback loops generate robust ~24 h cellular rhythms.

The mammalian circadian system operates as a sophisticated hierarchical network, with the suprachiasmatic nucleus (SCN) in the hypothalamus functioning as the master pacemaker (Figure 1) [103,104]. This master timekeeper coordinates peripheral clocks, which are found in virtually every tissue, throughout the body via multiple pathways, including hormonal signals, temperature fluctuations, and activation of the sympathetic and parasympathetic nervous systems [105,106,107]. This precise temporal organization ensures rhythmic coordination from the organismal level down to individual cells [108].

At the cellular level, circadian rhythms emerge from interlocked transcriptional–translational feedback loops (TTFLs) that generate self-sustained oscillations with approximately 24 h periodicity [109]. The primary feedback loop relies on the transcription factors CLOCK and BMAL1, which heterodimerize to activate transcription of *Period* (*PER*) and *Cryptochrome* (*CRY*) genes by binding to enhancer box (E-box) motifs in their promoter regions [110,111,112,113]. The resulting PER and CRY proteins subsequently form inhibitory complexes that suppress CLOCK-BMAL1 activity, thereby repressing their own transcription and establishing the core oscillatory mechanism [114]. Additional regulatory layers enhance robustness and enable fine-tuning of circadian timing. Secondary feedback loops involve the nuclear receptors REV-ERBα/β (encoded by NR1D1/2) and RORα/β/γ, which compete for binding sites in the BMAL1 promoter to provide additional transcriptional control [115,116]. Tertiary loops include transcription factors such as NFIL3 and DBP, which modulate ROR expression and contribute to the generation of distinct oscillatory phases [97]. This multilayered architecture creates a remarkably stable yet adaptable timing system capable of maintaining precise rhythmicity while responding to environmental and physiological perturbations [114,117,118,119,120,121,122,123].

### 2.1. Circadian Control of Cellular and Physiological Processes

The influence of circadian clocks extends far beyond the core clock network, with approximately 40% of genes exhibiting rhythmic expression patterns in a tissue-dependent manner [124,125,126,127]. This widespread transcriptional control enables circadian coordination of virtually every aspect of cellular physiology. Sleep–wake cycles represent the most familiar circadian output, mediated through rhythmic regulation of cortisol (promoting morning alertness) and melatonin (facilitating evening sleepiness) [128,129].

Beyond our sleep–wake cycle, metabolic processes are strongly regulated by the circadian clock. Core clock components BMAL1 and CLOCK directly control enzymes involved in glucose uptake, lipid metabolism, and energy homeostasis, while REV-ERBα and RORα modulate lipid and glucose metabolism [130,131,132]. These regulatory mechanisms manifest as daily fluctuations in hunger, energy levels, and metabolic efficiency that align with anticipated feeding and fasting periods.

Cell cycle control represents another critical intersection between circadian clocks and cellular physiology [133,134,135,136]. Clock-controlled genes such as WEE1, which inhibits mitotic entry, and p21, which governs the G1/S transition, ensure that cell division occurs in a temporally coordinated manner [137]. Additionally, PER and CRY proteins interact directly with cell cycle regulators, creating bidirectional communication between circadian timing and DNA replication machinery [137].

The circadian system orchestrates DNA repair processes through rhythmic expression of repair proteins like XPA, a key component of nucleotide excision repair [138,139]. This temporal coordination aligns DNA repair capacity with periods of heightened damage risk, such as during daylight exposure to UV radiation [139]. Beyond these fundamental processes, circadian clocks regulate immune responses, cognitive performance, cardiovascular function, and numerous other physiological processes, highlighting their central role in maintaining health and homeostasis [140,141,142].

### 2.2. Circadian Immune Regulation

The immune system is tightly organized by the circadian clock across both innate and adaptive responses, with important consequences for cancer immunosurveillance and immunotherapy efficacy [143]. Circadian regulation shapes immune function at multiple levels by controlling the trafficking and positioning of immune cells, tuning their activation state, and timing the production of cytokines and other mediators. These temporal programmes define when immune responses are most effective and when tumours may be more likely to escape detection.

Immune cell migration between tissues follows robust daily rhythms [144,145,146]. Dendritic cells, which coordinate innate and adaptive immunity, exhibit circadian patterns in their migration from peripheral tissues to lymph nodes [146]. Lymphocyte trafficking into lymph nodes is likewise under circadian control, regulated by rhythmic BMAL1-dependent expression of ICAM-1 on vascular endothelium [144,145]. The temporal convergence of dendritic cell arrival and lymphocyte availability determines the efficiency of antigen presentation and the magnitude of T cell priming. In humans, afternoon vaccination yields stronger immune responses, consistent with the timing of peak dendritic cell migration and lymphocyte infiltration [144,145]. Beyond dendritic cells, T cells and B cells show rhythmic trafficking and lymph node cellularity driven by clock-controlled chemokine receptors and adhesion pathways [143,145]. These rhythms create time-of-day differences in immune surveillance that could shape when tumour cells are more likely to evade immune control.

Within tumours, immune cell populations themselves oscillate in abundance and functional state. Tumour-infiltrating lymphocytes display time-of-day-dependent rhythms that shape the efficacy of immunotherapy [38]. In preclinical models, immune checkpoint blockade administered during periods of maximal lymphocyte infiltration leads to superior tumour control, and adoptive CAR T cell therapy shows enhanced efficacy when delivered at optimal circadian phases [38]. Immunosuppressive compartments are likewise rhythmic. PD-L1-expressing myeloid-derived suppressor cells peak at defined times of day within tumours, and anti-PD-L1 therapy is most effective when administered in synchrony with this peak, when target availability is highest [39]. These studies illustrate how aligning therapy with circadian immune dynamics can substantially improve anti-tumour responses and form a mechanistic basis for chronoimmunotherapy (also see Section 4).

The functional significance of circadian immune regulation is further demonstrated by disruption studies. Chronic perturbation of light–dark cycles accelerates tumour growth in murine models and abolishes or inverts normal daily patterns of macrophage polarization and cytokine production [40]. Under these conditions, the balance between proinflammatory M1 and immunosuppressive M2 macrophages is dysregulated, fostering a tumour-permissive TME. Adaptive immunity is also compromised, with impaired T cell priming and reduced cytotoxic activity [40]. In breast cancer, circadian misalignment reshapes the immune microenvironment and increases a cancer stem cell phenotype, further promoting immune evasion [35]. Epidemiologically, night shift work, a common cause of chronic circadian disruption, is associated with increased breast cancer incidence [90]. Experimental ablation of central clock function by chronically alternating light cycles similarly leads to faster mammary tumour growth in mice [95], with proposed mechanisms including impaired immune surveillance and MDSC recruitment via the CXCL5-CXCR2 axis. However, given the complex network of potentially mediating factors, including hormonal, metabolic, and immune pathways, disentangling the precise mechanisms by which circadian disruption promotes breast tumorigenesis remains a significant challenge.

Given the demonstrated impact of circadian rhythms on anti-tumour immunity, translational efforts are now underway to optimize immunotherapy timing. Meta-analyses of retrospective studies investigating the time-of-day of immunotherapy infusion on survival of patients with advanced cancers suggest that treatment timing can influence both efficacy and immune-related toxicity [147,148]. Although optimal schedules likely depend on cancer type, drug class, and patient chronotype [149], integrating circadian profiling into immunotherapy protocols represents a promising strategy to improve therapeutic index, particularly in breast cancer subtypes such as TNBC, where immunotherapy has been actively tested in clinical trials, but responses have been inconsistent [150,151,152]. This approach may be especially relevant in older patients, as ageing is accompanied by circadian dampening and loss of temporal immune gating, leading to chronic inflammation and impaired immune surveillance [153].

### 2.3. Circadian Clocks and Ageing

With ageing, circadian clocks lose amplitude and precision, and the number of rhythmically expressed genes in tissues declines [154,155,156,157]. Given the centrality of the circadian clock to the temporal organization of physiology, age-related weakening of circadian rhythms may be far more influential for human health and longevity than anticipated. For many years, the field has been aware that mice with mutations in clock genes show reduced life span and age-related diseases, including increased tumour incidence [158,159,160,161]. In humans, weakening of circadian clocks has been associated with increased prevalence of breast cancer, metabolic syndrome, cardiovascular disease, osteoporosis, and bone fractures [42,162,163,164,165]. Optimizing circadian rhythms through lifestyle interventions can restore rhythm robustness and mitigate age-related disease, including cancer [166,167].

Recently, time-restricted feeding (TRF) has gained attention as an intervention that counteracts ageing-related clock weakening. In fact, TRF has been shown to increase longevity via restoration of tissue-specific diurnal transcription, as well as inter-tissue circadian synchronization [168,169,170]. Strengthening circadian oscillations results in improved rhythmicity of key metabolic, immune, and autophagy pathways [168,169,170], implying circadian regulation contributes to healthy ageing. Interestingly, TRF intervention in breast cancer survivors also appears to lower the risk of recurrence, potentially by restoring circadian rhythms and temporal tissue homeostasis [171,172].

Nicotinamide adenine dinucleotide (NAD^+^), nicotinamide phosphoribosyl transferase (NAMPT), and sirtuins (e.g., SIRT1) have been implicated in circadian regulation by modulating clock protein stability, chromatin state, and nuclear translocation events important for robust circadian clock function [173,174,175]. Consistently, restoring NAD^+^ levels can reprogram circadian dynamics by promoting PER2 nuclear translocation, thereby counteracting aspects of circadian ageing [176]. Interestingly, NAD^+^ supplementation may also improve life span [177,178], as well as exert anti-tumour activity and suppress metastasis by activating the SIRT1-p66Shc axis in experimental models of TNBC [179]. Hence, NAD^+^ biosynthesis might be an important link between ageing, circadian clocks, and breast cancer.

Interestingly, the process of autophagy, a self-degradative process important for balancing energy sources, removal of misfolded/aggregated proteins, clearing of damaged organelles, and elimination of intracellular pathogens, loses circadian rhythmicity with ageing [156]. Autophagy can be cytoprotective, cytostatic, cytotoxic, or nonprotective in breast cancer [180]. It can function as a tumour suppressor through the clearance of damaged organelles and reducing oxidative stress, preventing tumour initiation, or it can support cancer cell survival under metabolic stress, sustain cancer cell stemness, and facilitate adaptation to hypoxia and nutrient deprivation [181]. The circadian disruption of autophagy associated with ageing might thereby promote tumour cell survival and growth, metastasis, and therapy resistance.

Immune responses that are normally gated by time-of-day also show weakened diurnal variation in aged organisms [153]. Loss of temporal gating contributes to a state of chronic, low-grade inflammation and simultaneously impairs the timing and efficacy of immune surveillance. Compromised circadian-mediated immune dysrhythmia may thereby permit beast neoplasia, enhance cancer growth, and metastasis (also see Section 2.2).

Lastly, it is also important that circadian clocks control aspects of ECM homeostasis, including rhythmic collagen turnover, as well as expression of signalling molecules and ECM-degrading proteases. Vice versa, the biochemical and biomechanical properties of the ECM influence circadian clock function. Therefore, it is important to know whether a cell type normally lives in a biomechanically soft or stiff environment, as changes in the naturally occurring mechano-environment will alter clock properties [182]. For example, there is evidence of age-induced perturbation of circadian clock regulation affecting the ECM [183] (also see Section 2.5). Ageing appears to be associated with perturbation of rhythmic ECM gene expression, collagen turnover, and collagen crosslinking, resulting in a stiffened, fibrotic stroma throughout multiple tissues within the body. This stiffened, fibrotic connective tissue in turn may compromise circadian rhythmicity further [54,182]. In 2017, Yang et al. report that ageing-related stiffening of the periductal ECM in mammary tissue is, similar to the stiffened stroma adjacent to a breast tumour, associated with reductions in the amplitude of epithelial circadian clocks compromising tissue homeostasis, stem cell rhythmicity, and cell growth [53,54,184]. These alterations in circadian clocks could account for data showing how a stiffened ECM fuels tumour heterogeneity, malignant transformation, and therapy resistance [6].

### 2.4. Circadian Clocks and Metabolic Disease

Clock gene/protein oscillations generate daily rhythms that coordinate energy and lipid metabolism, insulin secretion, and glucose homeostasis [160,185,186,187,188]. Circadian disruption, through lifestyle-driven or genetic modifications of circadian clock genes, alters metabolic setpoints and promotes the development of disorders, including metabolic syndrome, obesity, diabetes, and cancer [87,189,190,191].

Given that the clock is intimately involved in regulating metabolism, the intersection of cancer metabolism and its control by the circadian clock is an area of active investigation. Many cancer cells display increased levels of aerobic glycolysis, glutamine oxidation, lipogenesis, and nucleotide synthesis [192]. This raises the question as to whether the high metabolic demand of rapidly proliferating cells might be linked to circadian disruption and vice versa. Consistently, *Bmal1* knock-out fibroblasts have been shown to display heightened glycolysis, elevated lactate production, reduced lipid oxidation, and ATP production [193], suggesting circadian disruption promotes a metabolic switch toward glycolysis as has been observed in cancer cells. Glycolysis-derived lactate might similarly contribute to clock disruption under hypoxic environments, as has been observed within the hypoxic core of a tumour [194]. Importantly, depletion of NAD^+^ causes a shift toward glycolysis by impairing mitochondrial lipid oxidation, promoting lactate production from pyruvate, and attenuating the activity of sirtuins. Low levels of NAD^+^ can disrupt clock function [173,174] and favour aerobic glycolysis in cancer [192]. Consistently, genetic clock disruption in a Kras-LSL-G12S, p53 flox/flox mouse model potentiated glucose consumption and lactate excretion [195], implying that clock dysfunction can exacerbate tumour metabolism to support tumour development and progression [196].

Epidemiological evidence connecting circadian disruption by shift work with cancer has largely focused on hormone-dependent diseases such as breast and prostate cancer, which raises the possibility that additional clock-controlled endocrine factors may be at play. Melatonin, a sleep-regulating hormone under the control of the SCN central pacemaker clock, has been linked to mitochondrial oxidation, tumour lipid metabolism, and tumour-suppressive functions [197,198]. Interestingly, melatonin administration, which may strengthen circadian rhythmicity, appears to counteract tumour growth in the mammary gland under disrupted light–dark cycles [199,200,201], while inhibition of melatonin synthesis under shift work conditions may increase breast cancer risk [91,95]. Additionally, chronic shifts in light schedules promoting breast tumour growth have been reported to ablate circadian rhythms of glucocorticoid levels [95], which play a central role in anti-cancer immunity and stress response. Hence, reinforcing circadian rhythmicity through lifestyle interventions, such as maintaining consistent sleep schedules and light exposure patterns, may be beneficial for maintaining a tumour-suppressive metabolic state.

### 2.5. Circadian Clocks and Mechanobiology

In multicellular organisms, cells exist within a complex microenvironment composed of a cellular stroma and an ECM that provides biochemical and biomechanical cues directing cellular functions. Once considered merely structural, the ECM is now known to play a central role in regulating cell signalling, tissue development, and function. These effects are mediated through adhesion receptors that sense and transmit information about the surrounding physical and chemical environment. Disruption of ECM function contributes to age-related diseases and presents therapeutic opportunities for treating conditions such as cardiovascular disease, cancer, and impaired wound healing [6].

**Figure 2:**
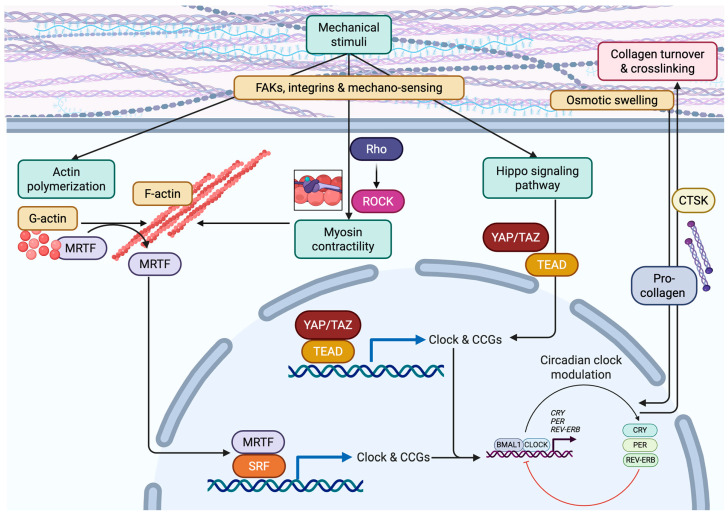
Biomechanical signals linking ECM and circadian clocks. Cell autonomous circadian clocks interact with their mechanoenvironment through cell–ECM interactions mediated by focal adhesion complexes, integrins, or other mechano-sensing pathways. Actin polymerization impacts rhythmic gene expression via MRTF nuclear translocation and co-regulation of SRF-driven transcription. Rho/ROCK-mediated myosin-dependent cell contraction further feeds into this pathway and promotes release and nuclear translocation of MRTF. Additionally, hippo pathway regulates rhythmic gene expression through nuclear translocation of YAP/TAZ and transcriptional activity via TEAD in response to stiff environments. Lastly, circadian clocks maintain rhythmic collagen homeostasis via rhythmic synthesis and secretion of pro-collagen, as well as rhythmic regulation of the proteinase CTSK. Likewise, mechanical loading and associated rhythmic changes in osmolarity act as bona fide entrainment cues for circadian clocks.

While circadian clocks are known to modulate biochemical circuits linked to cell signalling and gene transcription, links between circadian oscillations and mechano-signalling remain less clear. Nevertheless, reciprocal communication between cellular clocks and mechanobiological cues, cell–cell and cell–ECM force, and actomyosin tension in response to a stiff ECM and RTK signalling have been reported (Figure 2). In 2013, conducting an unbiased screen for immediate early transcription factors that regulate circadian dynamics, Gerber et al. identified serum response factor (SRF) as an important regulator of cellular clocks [202]. Since then, actin cytoskeleton remodelling and downstream transcriptional SRF activity in response to myocardin-related transcription factor (MRTF) has been described as an important mechanotransduction-stimulated output that feeds into the molecular clock machinery.

Focal adhesion and growth factor receptor/G protein-coupled receptor and noncanonical TGFb receptor-mediated stimulation of Rho-GTPase/ROCK activity alter actin cytoskeleton organization by regulating actin polymerization. Actin polymerization, in turn, increases filamentous actin to release MRTF-associated G-actin that permits its nuclear translocation and the activation of SRF-mediated transcription [203]. In 2022, Xiong et al. described how the integrin–actin cytoskeleton–MRTF/SRF pathway modulates cellular oscillations. They reported that pharmacological perturbation of actin polymerization and Rho GTPase signalling, or genetic perturbation of MRTF/SRF, disrupted circadian rhythmicity at the cellular level [204]. Blocking integrin–ECM interaction and associated focal adhesion signalling, as well as altering physical properties of the ECM by seeding cells into scaffolds with different stiffness, similarly altered circadian oscillations [204]. Consistently, Xiong et al. reported direct transcriptional control of circadian clock genes, specifically *Per2*, *Cry2*, and *Nfil3*, by MRTF/SRF. The findings suggest there exists a circadian clock mechanism that senses the local microenvironment and implicates integrin–ECM adhesions and matrix stiffness in this phenotype. Conversely, while circadian oscillations are regulated by actin dynamics downstream of RTK and integrin signalling, circadian clocks can also modulate actin polymerization, organization, and turnover. For example, Hoyle et al. report that cell-autonomous circadian clocks drive temporal proteomic programmes that impose rhythmic regulation upon the actin cytoskeleton [205]. Specifically, the authors observed a circadian rhythm in F:G actin ratio, without rhythmic total actin abundance. Moreover, this observed circadian regulation of actin dynamics translated into rhythmic regulation of cell migration, adhesion, and wound healing.

In addition to its impact on actin dynamics, the stiffness of the ECM can also exert a cell-type-dependent effect on circadian rhythms. In 2017, Yang et al. reported that increasing the stiffness of a tissue suppressed circadian clock activity in mammary epithelial cells in the gland [54]. These ECM stiffness effects on the molecular core clock machinery were mediated via integrin/focal adhesion signalling through activation of the Rho/ROCK pathway and actomyosin contractility. These findings raise the possibility that stiffness-dependent actomyosin cellular tension modulates rhythmic gene expression and thereby influences mammary tissue regeneration and tumorigenesis. A subsequent study by Williams et al. (2018) reported that a stiff ECM enhances circadian clock gene oscillations in stromal cells within the mammary gland, lung, and skin [52]. These findings suggest that local biomechanical tissue properties, such as ECM viscoelasticity and stiffness or locally applied strains and stretch, could simultaneously and differentially impact cell autonomous circadian clocks.

Mechanisms for why epithelial cells show differential circadian-dependent regulation in response to mechanical cues, such as a stiff ECM, remain unclear. However, one clue was provided by Abenza et al., who identified the YAP/TAZ-TEAD pathway as a key mechanosensory signal that modulates cellular circadian clocks. Specifically, they showed that fibroblast clocks can be disrupted by YAP/TAZ nuclear translocation [206]. Given that YAP/TAZ is transcriptionally activated in response to mechanical cues, including a stiff ECM [207], the enhanced stromal circadian rhythms observed in cells interacting with a compliant (soft) microenvironment may be related to nuclear levels of YAP/TAZ and its TEAD-dependent transcriptional activity [206]. Consistently, Francis and Rangamani used a computational model to investigate interactions between cell autonomous circadian clocks and cellular mechanotransduction mediated via the MRTF/SRF and YAP/TAZ pathways [208]. Their model predicted that YAP/TAZ and MRTF integrate distinct mechanotransduction signals, with G-actin dimerization decreasing nuclear YAP/TAZ levels and MRTF increasing nuclear YAP/TAZ.

It remains unclear which upstream signals transduce differential mechanosignalling to regulate circadian clocks in epithelial and stromal cells. Nevertheless, these effects are likely mediated through integrin specificity and actin crosslinkers [182], or via distinct biochemical cues such as serum-derived or tissue-resident growth factors [107,202,209,210]. Indeed, Chang et al. (2020) reported that cellular fibroblast circadian clocks maintain rhythmic collagen homeostasis via the secretory pathway [211]. Here, the synthesis and transport of pro-collagen are regulated via rhythmic expression of secretory pathway components at the endoplasmic reticulum, Golgi, and post-Golgi compartments. Likewise, rhythmic collagen degradation was achieved through circadian oscillations in the proteinase CTSK. In vivo and in vitro, circadian disruption led to an abnormal collagen matrix, with disturbed collagen fibre formation and collagen homeostasis. Consistently, Dudek et al. (2023) described a role for circadian clocks in mechanical loading in cartilage and intervertebral disc (IVD) tissue [212]. Their studies showed how hyperosmolarity, which is generated by mechanical loading and swelling pressure, activates the PLD2–mTORC2–AKT–GSK3b axis, which in turn resets circadian clocks and restores rhythmic gene expression. Perturbing cartilage and IVD circadian clocks resulted in an imbalance of anabolic and catabolic processes, subsequently leading to accelerated tissue ageing and degeneration.

Together, these findings emphasize the integration of mechanosignalling in regulating tissue homeostasis via its effects on the functioning of cellular circadian clocks.

## 3. Circadian Clocks and Breast Cancer

**Figure 3:**
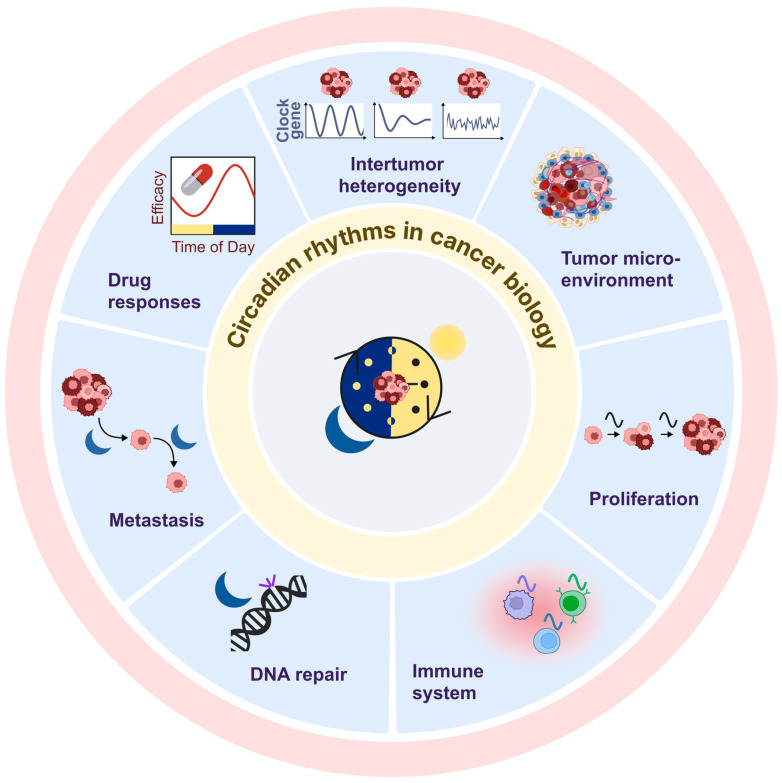
Circadian control of cancer hallmarks. Circadian rhythms shape multiple cancer hallmarks, including tumour cell proliferation, immune interactions, DNA repair, metastasis, and drug responses, with treatment efficacy varying by time of day. Circadian heterogeneity between tumours contributes to inter-tumour variability while tumour cell circadian clocks actively organize the tumour microenvironment by rhythmically regulating immune responses, stromal signalling, and extracellular matrix remodelling.

Circadian clocks have emerged as critical regulators of cancer initiation and progression (Figure 3). Disruption of circadian rhythms, through genetic, environmental, or behavioural factors, has been implicated in oncogenesis [195]. Initial observations linking circadian misalignment to cancer date back to the 1960s [213], with mounting epidemiological evidence supporting this connection across diverse tissues, including the breast [90,95]. For example, epidemiological studies revealed that women engaged in long-term night shift work consistently show elevated breast cancer risk, prompting the International Agency for Research on Cancer (IARC) to classify shift work involving circadian disruption as “probably carcinogenic to humans” [91,214].

Emerging evidence indicates that circadian rhythms modulate key steps in tumour progression and metastasis. For instance, circulating tumour cells (CTCs) disseminate into the bloodstream following a distinct circadian pattern rather than occurring at a constant rate [48]. CTC levels peak during the rest phase, which corresponds to nighttime in humans and daytime in nocturnal animals like mice. Notably, these rest-phase CTCs exhibit higher expression of genes related to cell proliferation and mitosis, and they display greater metastatic potential compared to those shedding during the active phase. These observations imply that specific circadian controls remain operative even in advanced tumours, creating temporal “windows of opportunity” during which cancer cells may be more prone to detach and colonize distant organs.

At the molecular level, breast tumours frequently exhibit altered expression of core clock genes compared to adjacent normal tissue, which reflects rewiring of circadian control mechanisms during tumorigenesis. Several studies have shown elevated levels of *CLOCK*, *CRY1*, and *RORγ*, and reduced expression of *CRY2*, *PER1*, *PER2*, *PER3*, and *RORα* in breast tumours compared to healthy tissue [34,92,93,94,215]. These gene-level changes were shown to have distinct functional consequences, given their involvement in multiple cellular processes central to oncogenesis. For instance, overexpression of *CRY1* leads to increased proliferation and reduced apoptosis, primarily through suppression of WEE1. The latter is a kinase that normally inhibits CDK1 activity to delay mitotic entry at the G2/M checkpoint [45,216,217]. Hence, loss of WEE1-mediated control allows cells to progress prematurely into mitosis, contributing to uncontrolled growth [218]. *CRY1* has also been linked to the Hippo pathway, which is a key signalling network controlling organ size through regulation of cell proliferation, apoptosis, and stem cell renewal. Notably, the Hippo pathway has been implicated in the mechanosignalling-dependent regulation of circadian clocks. Disruptions in the Hippo pathway can lead to excessive cell growth and impaired differentiation, further linking *CRY1* to cancer development and progression [45]. Elevated *RORγ* levels have been shown to play a key role in metabolic reprogramming of breast cancer cells, leading to the hyperactivation of the cholesterol biosynthesis programme that in turn supports the malignant phenotype [219]. By contrast, the downregulation of *CRY2*, *PER* genes, and *RORα* reduces their tumour-suppressive activity to foster a cancerous phenotype [215,220,221]. For instance, loss of CRY2 increases NF-κB pathway signalling to elevate cell growth and survival and alter autophagy regulation [222]. Similarly, reduced expression of *PER1* and *PER2* leads to disrupted interactions with cell cycle regulators such as *p21* and DNA damage response genes like *ATM*, *CHK2*, and *p53*, ultimately promoting tumour progression and reducing apoptosis [215,220,223,224]. Finally, the downregulation of *RORα* promotes an invasive phenotype, given the role of *RORα* in inducing the expression of the repressive microenvironmental factor *SEMA3* [221].

Beyond individual gene-level effects, global circadian regulation in breast cancer appears to be shaped by tumour subtype and disease stage, reflecting the broader molecular heterogeneity that characterizes breast cancer. Large-scale transcriptomic studies have shown that luminal-type tumours retain partial circadian rhythmicity, while basal-like and triple-negative breast cancers (TNBCs) often exhibit strongly dampened or arrhythmic expression profiles [34,94]. This underscores the considerable heterogeneity in circadian phenotypes even within breast cancer subtypes. Whether these perturbations in clock regulation reflect the distinct stromal TMEs of these breast tumours remains unclear. However, it is likely that they may be linked to the fact that more aggressive TNBCs and HER2^+^ breast cancers also develop the stiffest stroma [27].

In Lum A tumours, partial rhythmicity is retained in genes related to epithelial–mesenchymal transition (EMT) and cell invasion. Interestingly, stronger circadian amplitude in these tumours has been linked to worse clinical outcomes, suggesting that persistent circadian function in tumours can, paradoxically, temporally gate pro-metastatic programmes and drive disease progression in specific contexts [34]. This is conceptually distinct from evidence that systemic circadian disruption accelerates metastasis by impairing host immune surveillance [35]. Together, these findings illustrate that both the presence and absence of circadian rhythmicity can drive tumour progression depending on cellular context. While these findings highlight inter-subtype variability, recent preclinical work with various breast cancer cell lines has revealed intra-subtype circadian heterogeneity, particularly within TNBC [43]. Using high-resolution time-series recordings of two core clock genes, *Bmal1* and *Per2*, and computational modelling, the authors classified breast cancer cell lines into four circadian phenotypes: (1) functional clocks (stable ~24h oscillations), (2) weak clocks (low-amplitude rhythms), (3) unstable clocks (quickly desynchronizing rhythms), and (4) dysfunctional clocks (long periods and quick desynchronization of rhythms). Crucially, these phenotypes did not align strictly with clinical subtypes. Some TNBC lines (e.g., MDA-MB-468 and HCC1143) surprisingly retained strong rhythmicity, while others (e.g., HCC1806, MDA-MB-231) showed weak or unstable rhythms. This challenges the assumption that aggressive cancers like TNBC are uniformly clock-deficient and underscores that clock functionality can vary even within a single subtype, a variability that had previously been observed in other cancers, such as colorectal cancer, as well [225].

These findings suggest that circadian dysregulation in breast cancer is neither binary nor uniform but rather distributed along a functional spectrum shaped by subtype and intra-tumoral context. Circadian profiling may thus provide additional stratification beyond existing molecular subtypes, with potential diagnostic and therapeutic value.

### Circadian Clocks and Mechanobiology in Breast Cancer

Modern lifestyle, e.g., shift work, travel across time zones, “social jet lag”, and also ageing, frequently leads to disruptions in the circadian system. Vice versa, ageing and age-related diseases commonly lead to alterations in tissue architecture and stiffer mechanoenvironments, which contribute to perturbations of circadian oscillations and disrupted tissue organization (also see Section 2.3). In the breast, Yang et al. found ageing-related dampening of epithelial clocks as a consequence of stiffened periductal ECM that was mediated via increased cell–ECM integrin signalling [52,54,182]. Likewise, tumours normally have an increased stromal stiffness due to excessive deposition, increased remodelling, and elevated crosslinking of ECM components [6]. Cell-autonomous circadian clocks are fundamental in gating cell division, regulating DNA damage responses, and controlling cellular adhesion, migration, and metabolism. Therefore, mechanical microenvironment changes that alter daily fluctuations in clock target genes and tissue homeostasis may contribute to tumorigenesis [226].

Circadian gene expression has been observed in the mammary glands of various mammals, including mice and humans [42,53]. Beyond core clock genes, hundreds of other breast-expressed genes similarly follow a circadian pattern. Importantly, the altered expression of these genes can influence breast tissue structure and function to promote cancer [32,42,53]. Notably, mammographic density, one of the greatest risk factors for breast cancer, is associated with a physically stiffer stroma [227,228]. This is accompanied by elevated integrin-mechanosignalling as well as an expanded stem cell pool [30,68], suggesting that a stiffer stromal ECM contributes to malignant transformation. In 2018, Broadberry et al. reported that stroma adjacent to breast tumours is indeed stiffer than that of normal tissue within the same individual [53]. Moreover, they showed that the circadian clocks in tumour epithelia exposed to these stiffer environments become severely dampened, which is associated with uncontrolled cell growth [53]. Circadian clocks also play a key role in maintaining mammary stem cell function in response to the extracellular environment [54]. This is consistent with recent work showing how a stiffer stroma and elevated integrin mechanosignalling expand the stem/progenitor cell pool [30]. Hence, disruption of circadian rhythmicity might promote tumorigenesis via the alteration of stem cell activity and dormancy. In fact, mice with a disrupted BMAL1/CLOCK complex (such as the CLOCK Δ19 mutant) show impaired self-renewal of mammary progenitor cells [54,93]. Likewise, circadian clocks contribute to the rhythmic regulation of the mechanical microenvironment through modulation of collagen and ECM homeostasis [183,211], and circadian disruption has been associated with fibrotic disease in various tissues [229,230,231]. Hence, it is highly likely that chronic circadian disruption, as experienced by long-term rotating shift work or in ageing, could contribute to loss of ECM structural integrity and homeostasis, accelerate ageing, and predispose to disease. Lastly, the core clock gene CRY1 has been shown to form a transcriptional–translational feedback loop with the YAP/TAZ/TEAD pathway, resulting in YAP/TAZ hyperactivation and enhanced DNA damage upon CRY1 downregulation in breast cancer cells [45].

Ultimately, cell-autonomous circadian rhythms, via the interaction with their mechanoenvironment, contribute to development and progression of breast cancer through two major mechanisms [32,42,183]: (1) altered mechanosignaling (e.g., RhoA/ROCK, F:G actin, MRTF/SRF, YAP/TAZ) promoting disruption of cellular circadian rhythms, which is further related to abnormal proliferation, DNA damage response, migration and invasiveness, epithelial to mesenchymal transition, cellular metabolism, etc., as well as (2) circadian disruption perturbing ECM homeostasis, related to pathological changes in tissue stiffness and bio-mechanical/-chemical signals (e.g., collagen secretion/degradation, MMPs, TIMPs, TGFb) that can promote fibrosis and an accelerated “ageing phenotype”.

## 4. Circadian Medicine

Breast cancer treatment strategies typically involve surgical tumour removal and localized radiotherapy, complemented by appropriate systemic therapy dictated by the molecular profile and stage of the breast tumour. However, while genetic heterogeneity has long been recognized as a driver of treatment resistance, non-genetic factors clearly also play an important role in shaping therapeutic response [232,233]. Intratumor heterogeneity can impact the probability, timing, and mechanism of cell death, with subpopulations of cells potentially entering alternative fates such as senescence or quiescence that may facilitate later disease recurrence [232,233,234,235,236,237,238]. Indeed, recent studies emphasized the spatial heterogeneity of the breast tumour stroma, including its stromal stiffness and adhesion signalling [25]. Chronotherapy, a treatment approach trying to optimize the timing of therapies to enhance their efficacy and tolerability, is attempting to address this issue [239,240].

Given the profound influence of circadian clocks on cancer biology, a growing area of research aims at leveraging circadian biology to improve cancer prevention and treatment [239,241,242]. Notably, the current standard practice for administering chemotherapy (and many other treatments) is largely dictated by hospital daytime schedules, with little consideration for the patient’s biological time. While practical, this approach ignores daily fluctuations in drug metabolism, cellular sensitivity, and DNA repair capacity that could mean the difference between a treatment’s success or failure. Circadian cancer medicine seeks to identify when during the 24 h cycle a particular treatment would be most effective against the tumour and/or least harmful to normal tissues and then align the administration accordingly.

Broadly, circadian-based treatment strategies can be viewed from two complementary perspectives: the patient’s circadian rhythm and the tumour’s circadian rhythm. A patient-centric strategy aims to minimize normal tissue toxicity by delivering therapy at the time of day when healthy cells are least susceptible to harm. In practice, this means timing drug administration according to the patient’s internal clock (chronotype) so that peak drug exposure coincides with the highest tolerance in healthy organs [243,244]. Accurate assessment of a patient’s circadian phase is therefore critical in this approach [239]. To address this issue, molecular biomarkers are being developed to estimate circadian phase from blood or saliva samples [243,244,245,246,247]. Once a patient’s rhythm has been characterized, it would be possible to design interventions to strengthen or resynchronize their circadian system before and during therapy. Strengthening a patient’s overall rhythmicity would help to restore normal homeostasis and widen the therapeutic window (the gap between tolerable dose and effective dose). Examples of circadian interventions include bright light therapy (to reinforce day-night cues), TRF schedules, melatonin supplementation, exercise routines, and emerging pharmacological agents that directly target clock components to adjust timing [109,129,248,249,250,251,252].

A tumour-centric circadian strategy focuses on exploiting any rhythmic vulnerabilities of the cancer cells themselves. There is evidence that cancer cells can harbour functional circadian clocks or oscillatory programmes of their own (even if altered from the normal state) [34,225,253,254]. If a tumour’s cellular proliferation, metabolism, or DNA repair capacity oscillates over time, then there may be specific times of day when the cancer cells are intrinsically less able to handle a cytotoxic drug or DNA damage, in other words, moments when the tumour is “weakest” [242]. Circadian cancer medicine seeks to identify these timepoints to maximize treatment impact on the tumour. However, implementing tumour-centric chronotherapy is challenging because each tumour’s clock can be unique [34,43,225]. This difficulty underscores the need for personalized chronotherapy schedules, which can be informed by laboratory testing of a patient’s own tumour [34,255,256]. Although this approach is still in early stages and resource-intensive, it holds promise: knowing a tumour’s clock could allow clinicians to schedule drugs at times when cancer cells are most likely to be cell-cycle arrested, DNA-damage-prone, or otherwise susceptible to therapy, all while the patient’s normal tissues are relatively protected.

While the interactions between circadian clocks and cellular TME components for shaping temporal dynamics in cancer immunotherapy are gaining attention (see Section 2.2 or detailed reviews here: [151,257]), the impact of changes in non-cellular TME components for chronotherapy has rarely been explored. We can speculate that fibrotic stroma and altered mechanoenvironments could blunt or reshape chronotherapeutic windows by modulating drug delivery, immune infiltration, and circadian functions of the cellular TME compartment. Circadian clocks modulate immune cell quantity, function, migration, and infiltration in the TME [38,39,148,258]. Therefore, a fibrotic stroma could hamper the efficacy of cancer immunotherapy by disrupting cell-autonomous circadian clocks through cell–ECM signalling or by providing a mechanical barrier, ultimately preventing optimal synchronization of immunotherapy administration with endogenous rhythms of crucial immune functions. Additionally, dense, collagen-rich ECM produced by activated stromal cells may reduce tissue perfusion, which would alter the optimal timing of drug delivery to tumour cells relative to systemic drug exposure. Moreover, fibrotic niches are often hypoxic and metabolically reprogrammed, which can contribute to circadian disruption of the tissue. Similarly, changes to the mechanoenvironment would perturb cellular circadian clocks, which in turn further promote pro-fibrotic changes to the stroma, creating a negative feedback loop that severely complicates both patient- and tumour-centric circadian treatment approaches.

## 5. Conclusions

The interplay between circadian clocks and the tumour microenvironment is a rapidly evolving and clinically important frontier in breast cancer research, acting as a double-edged sword, influencing disease risk and progression and offering therapeutic opportunities. Dysregulation of cellular and systemic circadian rhythms, e.g., due to stress, metabolic disease, ageing, or shift work, dampens clock amplitude and alters the breast cancer TME. Specifically, circadian disruption can weaken temporal immune gating and promote chronic inflammation that impairs anti-tumour immunity. In parallel, it drives abnormal epithelial cell proliferation and metabolic cycling, and reprograms stromal cells to remodel the extracellular matrix, thereby collectively fostering tumour growth, metastasis, and therapy resistance. Conversely, leveraging this rhythmic sensitivity provides novel therapeutic avenues, such as optimizing chronotherapy for improved drug efficacy and targeting specific clock–TME feedback loops to re-sensitize refractory tumours.

Despite these advancements in disentangling the relationship between circadian rhythms and the TME in breast cancer, critical contradictions and limitations in the field warrant careful consideration. Conflicting findings regarding the precise prognostic utility of core clock gene expression across different breast cancer subtypes likely reflect the reliance of most studies on bulk transcriptomics, which obscures cell-type-specific and spatially resolved rhythmicity. Similarly, the relative contribution of host- versus tumour-intrinsic circadian disruption to TME plasticity remains poorly defined. Addressing these open questions will require time-series single-cell and spatial omics approaches capable of capturing rhythmic molecular shifts within distinct TME compartments. Furthermore, future research should prioritize investigating how specific circadian perturbations dictate immune and stromal cell recruitment and functional states. In parallel, developing predictive methods to assess circadian state in breast cancer patients will be essential for enabling personalized, rhythm-optimized cancer treatments.

Key open questions include the following: (i) how clock strength, phase and cell-type specific rhythms differ across breast cancer subtypes and stromal niches; (ii) the precise molecular mechanisms by which ECM mechanics entrain or dampen local clocks in malignant versus normal cells; (iii) how TME dynamics should be incorporated into circadian medicine approaches; and (iv) how patient-specific factors such as chronotype, age, sex and comorbidities could be integrated to personalize chronotherapy. Addressing these questions will require integrative studies spanning molecular chronobiology, mechanobiology, and immunology, alongside clinical trials designed to test time-of-day-dependent interventions.

## Figures and Tables

**Table 1:** Important publications on the interconnection between circadian clocks and tumour microenvironment in (breast) cancer.

Author	Year	Key Findings
Ector, C. et al. [43]	2025	Deep circadian phenotyping across a panel of breast cancer cell lines shows 4 distinct circadian-based phenotypes—functional, weak, unstable, and dysfunctional clocks—that shape the response to anti-cancer drugs.
Li, S.-Y. et al. [34]	2024	Machine learning reconstruction of circadian timing from large patient datasets reveals subtype-specific clock reprogramming in luminal A breast cancer. Stronger tumour rhythmicity associates with reduced survival and enhanced EMT cycling, linking circadian activity to invasion and metastasis.
Wang, C. et al. [38]	2024	Quality and quantity of tumour-infiltrating lymphocytes, especially CD8+ T cells, exhibit circadian oscillations. The efficacy of chimeric antigen receptor T cell therapy and immune checkpoint blockage can be improved by adjusting the time of treatment. [*not breast cancer specific*]
Sun, C. et al. [44]	2024	Two circadian-related subtypes of breast cancer were identified that differ in their prognosis, clinical characteristics, and tumour immune microenvironments. Individuals with a high circadian-related risk score have a greater burden of tumour mutations, richer immune cell infiltration, and higher expression of immune checkpoint genes.
Azzi, A. et al. [45]	2023	The circadian clock gene *CRY1* is a direct target of the Hippo pathway effectors YAP and TEADs. In breast cancer cells, *CRY1* downregulation causes YAP/TAZ hyperactivation and enhanced DNA damage.
Dong, X. et al. [46]	2023	The circadian gene *TIM* enhances PD-L1 transcription and facilitates the aggressiveness and progression of breast cancer. *TIM* might play an immunosuppressive role in breast cancer and is inversely associated with CD8+ T lymphocyte infiltration.
Xiong, S. et al. [47]	2023	Low circadian rhythm signature of breast cancer is characterized by enriched immune-related pathways, immune cell infiltration, and anti-tumour immunity, while high circadian rhythm signature is associated with immunosuppression, synaptic transmission pathways, EMT, poor prognosis, and drug resistance.
Diamantopoulou, Z. et al. [48]	2022	Breast circulating tumour cell (CTC) dissemination and intravasation follow a circadian pattern, with rest-phase CTCs being prone to metastasize (upregulation of mitotic genes), whereas active-phase CTCs are devoid of metastatic ability. Circadian rhythm hormones, such as melatonin, testosterone, and glucocorticoids, dictate CTC generation dynamics.
Don, S.S.L. et al. [49]	2022	Conditioned media from breast cancer cell lines alter circadian rhythms of RAW 264.7 macrophages. Media from highly aggressive 4T1 cells caused loss of rhythmicity, while media from less aggressive EMT6 cells yielded no changes.
Shaashua, L. et al. [50]	2020	Expression of the core clock gene *Per2* in stromal cells is crucial for tumour initiation and metastatic colonization. Loss of *Per2* in the TME may contribute to a tumour-suppressive microenvironment. [*not breast cancer specific*]
Hadadi, E. et al. [35]	2020	Chronic jetlag-induced circadian disruption increases breast cancer cell dissemination and lung metastasis, enhances the stemness and tumour-initiating potential of cancer cells, and creates an immunosuppressive shift in the tumour microenvironment (CXCR2-mediated).
Aiello, I. et al. [40]	2020	Circadian disruption drives tumour-associated immune cell remodelling that facilitates tumour growth, including loss or inversion of M1/M2 macrophage polarization and altered cytokine levels. It further promotes cell proliferation while dampening the expression of cell cycle inhibitors. [*not breast cancer specific*]
Wang, J. et al. [51]	2019	BMAL1 overexpression enhances cell migration and invasion in breast cell lines. Functionally, it increases the expression and function of MMP9 and activates the NF-κB signalling pathway.
Williams, J. et al. [52]	2018	A stiff extracellular matrix dampens the oscillations of the epithelial molecular clock in mammary epithelial cells, while fibroblasts from the same tissue show the opposite response with strengthened and prolonged rhythmicity.
Broadberry, E. et al. [53]	2018	Circadian clocks are suppressed in primary breast cancer tumour cells in comparison to normal surrounding epithelia, which correlates with increased tissue stiffness around the tumour region.
Yang, N. et al. [54]	2017	The breast epithelial clock is regulated by mechano-chemical stiffness of the microenvironment in primary cell culture. The mammary clock is controlled by the periductal ECM in vivo, contributing to dampened circadian rhythm during ageing (via Rho/ROCK pathway).

## Data Availability

No new data were created or analysed in this study. Data sharing is not applicable to this article.
